# “It's like being involved in a car crash”: teen pregnancy narratives of adolescents and young adults in Jos, Nigeria

**DOI:** 10.1093/inthealth/ihab069

**Published:** 2021-10-18

**Authors:** Comfort Z Olorunsaiye, Hannah M Degge, Tina O Ubanyi, Timothy A Achema, Sanni Yaya

**Affiliations:** Department of Public Health, Arcadia University, Brubaker Hall, 450 S Easton Road, Glenside, PA 19038, USA; Department of Health and Education, Coventry University, Ashburn Road, Off Valley Road, Scarborough, YO11 2JW, UK; Department of Community Medicine and Primary Healthcare, College of Medicine and Health Sciences, Bingham University, Abuja-Keffi Express Way, PMB 005, Karu, Nasarawa State, Nigeria; Department of Community Medicine and Primary Healthcare, College of Medicine and Health Sciences, Bingham University, Abuja-Keffi Express Way, PMB 005, Karu, Nasarawa State, Nigeria; School of International Development and Global Studies, Faculty of Social Sciences, University of Ottawa, 120 rue Université privée, Ottawa, ON K1N 6N5, Canada; The George Institute for Global Health, Imperial College London, 84 Wood Lane, London W12 0BZ, UK

**Keywords:** adolescents, Nigeria, pregnancy, sexual and reproductive health

## Abstract

**Background:**

Adolescent pregnancy has serious public health implications, with far-reaching outcomes extending past the mother and child and affecting society. The purpose of this study was to explore the lived experience of adolescent pregnancy in Jos, Nigeria.

**Methods:**

We conducted in-depth interviews with 17 adolescents and young women ages 16–24 y in Jos, Nigeria who had experienced at least one teenage pregnancy. Participants were purposively recruited; each provided written informed consent before interviewing. We identified codes and themes using an inductive analytic approach.

**Results:**

Among the 17 participants, 14 had never been married and 10 had completed senior secondary school. Participants commonly associated adolescent pregnancy with inappropriate behaviour, immaturity and premarital childbearing. The main risk factors for adolescent pregnancy were lack of sexual and reproductive health education and parental communication. Pregnancy evoked feelings of fear, shame, anxiety and depression. Most pregnancies resulted in live births, while some participants had stillbirths or induced abortion. Some participants successfully completed their education post-pregnancy.

**Conclusions:**

Adolescents in this study lacked adequate sexual and reproductive health education that could empower them to make informed decisions and take action regarding their sexual and reproductive health. Multifaceted actions to address reproductive health education gaps can contribute to reducing adolescent pregnancy in Nigeria.

## Introduction

In 2019 there were 1.2 billion adolescents and youth ages 15–24 y, representing 16% of the global population.^1^ Adolescence, the period during which children transition to adulthood, can be a turbulent time,^2^ as they are faced with the pressure to grow up fast and also deal with displeasure from their peers, parents or caregivers.^3^ Annually about 21 million adolescent girls in low- and middle-income countries become pregnant.^4,5^ This often comes with dire consequences. Both the adolescent mother and her neonate have increased mortality rates. The risk of health complications, such as pre-eclampsia for the mother and low birthweight for the neonate, is elevated in adolescent childbearing.^6^ Additionally, adolescents who become pregnant are at increased risk of unsafe abortion and repeat adolescent pregnancies and many others experience obstructed and prolonged labour, both of which are leading causes of maternal morbidity and mortality.^7,8^ Moreover, many young mothers who survive obstructed labour may develop obstetric fistula and other disabilities.^9^ It is also documented that teenage mothers are more likely to be poor and unemployed and are less likely to complete secondary education and to proceed to higher education, thereby perpetuating the cycle of poverty and social disadvantage.^7,10^

With about 200 million people, Nigeria is the most populous country in Africa and has one of the highest absolute numbers of youth globally. Nearly 52% of the population is <18 y old.^11^ The median age at sexual debut is 17.2 y. About 43% of women ages 25–29 y were married before their 18th birthday, while 8% of girls ages 15–19 y were married by age 15.^11^ A study in a rural community in South West Nigeria reported the prevalence of adolescent pregnancy as 22.9%.^12^ This finding is similar to that of another study in Jos, North Central Nigeria, that reported a teen pregnancy prevalence of 25.5%.^7^ These results from localized studies highlight a higher prevalence of adolescent childbearing than the 19% national average^11^ and underscore the burden of teenage pregnancy in Nigeria.

Risk factors for adolescent pregnancy in Nigeria include poor information on sexual and reproductive health (SRH), peer pressure, sexual assault and rape, social media influence, poverty, poor access to contraceptives and cultural factors such as early marriage, among others.^13–15^ Regardless of the cause, adolescent mothers face social problems that have a profound impact on their lives. Such problems include discontinuation of her education, unsafe abortion, poverty, repeat teen pregnancies, exclusion from family and friends and an increased tendency to be involved in criminal activities.^7,16,17^ These stressors worsen future outcomes for the adolescent mother and her child, causing them to navigate the confines of poverty and increase their risks for mental health issues.^17,18^ In 2003 the Nigerian government launched the Family Life and HIV Education (FLHE) curriculum for junior secondary schools.^19^ The curriculum includes six key themes: human development, personal skills, sexual health, relationships, sexual behaviour and society and culture. Although the curriculum is recommended for teaching in schools, the implementation varies across states, thereby missing opportunities to educate and empower teenagers for informed reproductive decision making and SRH service use.^20^ For the adolescent mother to be a productive member of society, as she matures and fits into her role as an adult, there should be support within her close circle, the community and society, irrespective of her age and socio-economic position.^16,21^ There is a need to understand, in nuanced terms, how the adolescent pregnancy experience affects the immediate outcomes and achievement of short-term goals of youth in Nigeria from their own perspective. This study contributes to the field of adolescent and youth SRH by exploring the adolescent pregnancy experiences of young women, years after the experience. The key research question guiding the study was, ‘What are the perceptions and experiences of young people in Nigeria who had an adolescent pregnancy’? The results provide a nuanced understanding of adolescent pregnancy, through their own narratives, and reveal the initial decisions and the process of coming to terms with their situation, and importantly, how these experiences have influenced their social outcomes several years later. The findings may contribute to policies and programs to address the multidimensional risk factors for teenage pregnancy and to identify ways of empowering youth for informed reproductive health decision making and improved well-being.

## Methods

### Study context

Plateau State is one of the seven states in the North Central geopolitical zone of Nigeria. The state is diverse and inhabited by people of different ethnic groups, cultures and religions. Plateau State has an estimated population of 3.5 million people, approximately half of which is young people <20 y of age.^11,22^ The overall prevalence of teen births in Plateau State is 8.2%.^11^ However, a cross-sectional study of 13- to 19-year-old female residents of Jos^7^ found that one-fourth of the sample was ever or currently pregnant. Thus we conducted this study in Jos, the capital city of Plateau State in North Central Nigeria.

### Research design and participant selection

This was a qualitative study implemented using a phenomenological approach to gain a nuanced understanding of the lived experience of adolescent pregnancy. The data were collected from October to November 2016 using an in-depth interview guide. The population of interest for this study included adolescents and young women ages 16–24 y who had experienced an adolescent pregnancy, regardless of their marital status and parity, and who resided in Jos. We purposively recruited a convenience sample, initially from the teen crisis centre of the Bingham University Teaching Hospital, a faith-based, tertiary institution in the city. In addition to youth-friendly services provided in the hospital, the teen crisis centre provides counselling and SRH education to young persons. Further, we used snowball-sampling techniques to expand the sample by asking study participants to refer other young adults who met the study's inclusion criteria. We also solicited the help of personal and professional contacts in identifying other participants who did not use the services of the crisis centre. Our findings were reported based on the Consolidated Criteria for Reporting Qualitative Research (COREQ).

### Data collection

Four trained research assistants, comprising two doctors and two hospital counsellors, collected the data. The research assistants were trained in using probes appropriately during interviewing. The interviews were conducted in English at the teen crisis centre or another location chosen by the participants and by phone. Each interview lasted approximately 60 min. One participant did not complete the interview due to the emotional toll it had on her. Overall, there were 17 interviews included in the analysis. Past qualitative research on women's reproductive health in West Africa achieved data saturation within the first 12 in-depth interviews.^23^

### Data analysis

Interviews were transcribed verbatim and compared with the recordings for accuracy. We used an inductive thematic approach for the analysis, following Nowell et al.’s^24^ thematic analysis guide for rigorous and trustworthy qualitative research. Thematic analysis is a pragmatic way of summarizing qualitative data to produce an organized report of findings.^25^ Two of the study authors (CZO and HMD) analysed the data independently. Upon repeated reading of the transcripts, data analysis began with notetaking on ideas related to the study's purpose. Emerging codes, reflecting patterns and similarities from the data were iteratively identified. The researchers responsible for the data analysis compared and harmonized the initial codes and jointly broadened the codes to form categories, which were further developed into themes and subthemes. Where there were differences in data interpretation, both researchers met to discuss and resolve the differences. The codes and themes were shared with the other members of the research team for confirmation and validation. The findings were revised based on the feedback and insights of the research team to reach a consensus. The final themes and subthemes were jointly named by the research team. Representative quotes, reflecting the themes and subthemes, were selected for inclusion in the findings.

### Trustworthiness

For research to have an impact on policy and practice there must be evidence of methodological rigor and credibility of the findings.^24,26^ Rigor refers to ways of establishing trust or confidence in the findings of qualitative research. For the sake of quality management, from the outset, the purpose of the research was clearly defined and the authors and research assistants were integrated into the study's objectives. Moreover, an independent party, not involved in the study, transcribed the interview data. Multiple researchers carried out the data collection and analysis. TOU and TAA and two other trained research assistants were involved in the data collection. In addition, CZO and HMD worked independently to generate codes and thereafter, through consensus, refined the proposed codes and thematic patterns. TOU and TAA provided further clarification and insights into the findings.

To establish referential adequacy,^27^ the authors checked the preliminary findings with the raw data. Further, we sought feedback from two senior researchers in the field of SRH in sub-Saharan Africa with experience in qualitative research. Their feedback served as an external audit to boost the dependability of our findings. Equally, there were efforts to ensure methodical accuracy in the process of documenting and transcribing data details, backed by frequent cross-checking of the data and interpretations. Multiple informants and a rich description of the participants’ social and demographic characteristics have been provided to guide transferability.

### Ethical considerations

The Health Research and Ethics Committee of the Bingham University Teaching Hospital approved the study. In Nigeria, young people ≥16 y of age do not require parental consent but must consent themselves to participate in SRH research.^28^ Thus participants provided written informed consent before interviewing. They also granted permission to be audio recorded. Because it could be uncomfortable to relive some of the experiences elicited, we made it clear to the participants, in the informed consent, that they could opt out of answering any questions and/or stop the interview at any time; referral information for the crisis centre was also provided. All interview recordings and transcripts were de-identified and pseudonyms were used instead of participants’ names. The research data are accessible only to the research team. Each participant received an incentive, in the form of a mobile phone recharge credit for their preferred network, at the end of the interview as compensation for their time.

## Results

### Sociodemographic characteristics of study participants

Table 1 shows the sociodemographic characteristics of the study participants. At the time of data collection, the participants’ ages ranged from 16–24 y. Three participants were pregnant during data collection, 14 had never been married and 3 were separated or divorced. Ten participants had completed senior secondary education, while four had completed junior secondary school and three had completed tertiary education. All participants self-identified as Christian. An equal number of respondents, seven each, were employed or full-time students; three were unemployed. Six each of the respondents were either living with their parents or with others such as boyfriends and relatives, while five lived alone.

**Table 1.** Sociodemographic characteristics of the respondents

Characteristics	n	Values
Age (years), mean±SD	17	21.33±2.66
16–19	6	35.3%
20–24	11	64.7%
Age at first pregnancy (years), mean±SD	17	17.3±1.30
Pregnancies, mean±SD	21	1.24±0.79
Religion		
Christianity	17	100.0%
Marital status		
Never married	14	82.3%
Separated/divorced	3	17.7%
Highest level of education completed		
Junior secondary	4	23.5%
Senior secondary	10	58.8%
Tertiary	3	17.7%
Employment status		
Employed	7	41.2%
Unemployed	3	17.6%
Full-time student	7	41.2%
Living arrangement		
Lives alone	5	29.4%
Lives with parents	6	35.3%
Lives with others	6	35.3%

SD: standard deviation.

### Reflections on the meaning of adolescent pregnancy

Participants described teenage pregnancy as meaning different things to them, using such phrases as ‘terrible’, ‘not good’, ‘trauma’, ‘like being involved in a crash’ and ‘[pregnancy] at a young age’. Other descriptions of the phenomenon included ‘having a child out of wedlock,’ ‘giving birth while still at [parent's] home’, ‘not the right time’ and ‘like a child having a child’. Teenage pregnancy was also associated with inappropriate behaviour, being pregnant at an immature age and out of wedlock.

It means you are not up to have a child, yet you have it…It's just…you know that you're not adult, and you're not a child, when something happens before you have the sense of…maybe I could have done this, because then you are not up to that stage of even thinking about getting pregnant. [Mary; pregnant at 17 years; currently single, completed secondary education]

It is getting pregnant at the wrong time or out of wedlock…or getting pregnant at a young age…that is the wrong time to be pregnant…that person is not ready to be a mother. [Rifkatu; pregnant at 15 years; currently single, undergraduate]

None of the participants described adolescent pregnancy as a positive occurrence; their narratives reveal just how negatively they perceived the experience of teenage pregnancy.

…what I mean by real terrible is…to me, is that when I found myself with pregnancy, I was telling myself “no this is not true, and this is not how I planned my life”. It has changed all the plans I planned for myself, it made me start afresh. So it destroyed all what I [was] planning to do, and what I mean by terrible is that it gave me more pains. [Jummai; pregnant at 16 years; currently single, undergraduate]

Other participants described their experiences as traumatic.

[It] is like being in a car crash, actually, you don't know that you will be…you don't know that the car will crash, and you are inside the car, and suddenly, the car crashed. You just feel something that way, because you never expected that something will happen to you, and it now happened. [Rifkatu; pregnant at 15 years; currently single, undergraduate]

Ok, to me…I'll just put it in as…just a single word I'll use to describe it, trauma…That's how I'll just put it because…I don't know, it may not be like that, but in my case, that's what I experienced, and for a long time, which could have developed into something else, but I thank God. [Rose; pregnant at 16, 17 and 21 years; currently single, undergraduate]

### Unpacking the adolescent pregnancy journey

This theme encompasses participant's reflections on the factors that contributed to their pregnancy, discovering they were pregnant, their initial thoughts and reactions to the reality of being pregnant, the decision to keep or terminate the pregnancy and resilience in the face of difficulty. The subthemes are described below.

#### “Pregnant! How come?” Factors contributing to adolescent pregnancy

A lack of parent–child communication about SRH contributed to the occurrence of adolescent pregnancy.

…parents should open up to talk about sex with their children, especially in the teenage [years]. Let them…not just saying “don't allow a man to touch you”, but to tell her or tell him what will happen…they should not feel shy to talk to them, and…they [adolescents] will understand. Children will understand better when parents open up to tell them [about] what really happens, not just saying “don't touch”.…Don't touch does not mean anything. [Mary; pregnant at 17 years; currently single, completed secondary education]

There are things parents or guardians of adolescents should tell them about, how their body is changing, what they can do and what they cannot do, so that they don't feel that they are mature and can do whatever they like. [Patricia; pregnant at 17 years; currently single, undergraduate]

There was a dearth of SRH knowledge and information. Some participants, even when they had missed their period, did not realize they were pregnant. Rather, they interpreted the missed period as a sign of a sexually transmitted infection (STI). Consequently some were up to 5–6 months gestation before they realized they were pregnant.

Ok, erm…first of all, I had this…this toilet infection [STI], so I thought it was the infection…because they told me that the…if it's not treated immediately, that it would block my menstrual period, so I thought it was infection, and my mom too, she thought that it was it [infection]. It was when it was 6 months that I later discovered that I was pregnant. [Fatima; pregnant at 17 years; currently single, completed secondary education]

Well, I found out I was pregnant…when it was 6 months. I went to the hospital and…the doctor…I told him that there was something walking in my stomach, so he should check whether it's worms or something [else], but he just ran a test, he told me that I was pregnant. I did not believe it, I couldn't believe it. I just walked away. I told him that he [was] lying. [Jummai; pregnant at 16 years; currently single, undergraduate]

Although participants were sexually active, most were not actively using SRH services. There was widespread ignorance about pregnancy risk.

He was…I wasn't taking contraceptives… I didn't really know anything…much…I never really made inquiries about contraceptives…[I] did not have enough information about contraceptives and how to prevent pregnancy. [Hannatu; pregnant at 15 and 18 years; currently single, completed university education]

…Because based on my age, I was not thinking something like that [pregnancy] could happen. [Kaneng; pregnant at 17 years; currently divorced, post-secondary education]

That was my first time having sex and I didn't have any idea what to use or what I could do to protect myself. [Rifkatu; pregnant at 15 years; currently single, undergraduate]

Three cases of rape resulted in pregnancy.

They were brought to train us…to train the band on music and it was in the course of getting the materials for the band. It was from there that it happened…it was a normal conversation…and I was offered a drink by him…I took it and that was all that happened. There was not a drag [discussion] between him and me. [Monica; pregnant at 17 years; currently single, completed secondary education]

…In my case, they came and met me in my own domain; I did not go anywhere…It was forced on me to have the pregnancy that I did not want. [Patricia; pregnant at 17 years; currently single, undergraduate]

Contraceptive knowledge was poor; for some participants, the only way they knew they could prevent pregnancy was abstinence. Two participants recalled their partners advised them to drink salt water after sex as a home remedy to prevent pregnancy.

…and the only way to prevent pregnancy at that time is not having sex, that's all. We avoided…we prevented pregnancy by avoiding having sex. [Kemi; pregnant at 18 years; currently single, undergraduate]

I don't know because, that time we…I met [had sex with] him, he said that I should take salt and…that I will not be pregnant…If he tells me, I use to take it. [Rahila; pregnant at 16 years; currently separated, completed junior secondary school]

Although a few participants knew at least one modern contraceptive, they were unable to explain why they were not actively using any. In some cases, participants said their partner was using a condom or practicing withdrawal. Others recalled their partners opposed condom use, because condoms made sex less pleasurable, and insisted on unprotected sex. One participant thought condoms were only useful in preventing STIs. Related to this, some participants mentioned having ‘trust’ in their partners and/or themselves as an explanation for contraceptive non-use.

Condoms…No, I did not know about those ones [pills]…it's just condoms I knew. Then, it was not even about pregnancy, I just thought about not getting any type of disease [STI], so all we did was just go for tests, that's all…I [did] not even think of pregnancy. [Mary; pregnant at 17 years; currently singe, completed secondary education]

…but they [he] told me that he was not enjoying it with condom and that he don't use to release inside…but I think he was just lying because…I didn't know [then] that he was lying. [Naomi; pregnant at 16 years; currently single, completed junior secondary school]

Ok, because we trust each other so much, that kind of thing, it was not…was something that was based on trust, as in we just…we never used any protection, it was just him…it was just him. [Rose; pregnant at 16, 17, and 21 years; currently single; undergraduate]

#### The emotional toll of adolescent pregnancy

For the participants, experiencing adolescent pregnancy was a mix of emotions provoked by the imminent changes in their circumstances. The initial emotions described included fear, self-condemnation and guilt about shaming their family. There were also recurring narratives related to anxiety about coping, having to drop out of school and facing harsh punishment from the family.

…the time I discovered that I was pregnant, I was not happy, my thinking was…if my parents know that I was pregnant, what will they say? I'll bring shame to the family. [Kaneng; pregnant at 17 years; currently divorced, post-secondary education]

I felt like I had let down my younger ones who were looking up to me for example…The only fear I had was of my dad…I was afraid of the way he might react or take it…They [the school] would have expelled me and made me a public example. [Rifkatu; pregnant at 15 years; currently single, undergraduate]

I fear[ed] a lot…my fear was that…what will people say about me. I could not imagine me carrying a baby. That is my fear, because…I didn't have anything, I couldn't take care of the baby…how people will look at me. [Kemi; pregnant at 18 years; currently single, undergraduate]

For some participants, self-condemnation and anxiety about how their family would possibly react to the news triggered emotions that bordered on depression.

I was disappointed in myself. I felt very bad, as in, I regretted everything. I felt like dying because I just felt like life had stopped, so I didn't feel like doing anything to move on with my life. I just felt like that was the end of the world, I just felt very bad, like what will I tell my parents…all those things…I felt very bad, very disappointed. [Mary; pregnant at 17 years; currently single, completed secondary education]

I felt very bad. I just felt like…just ending my life. I even felt ashamed of myself. [Rifkatu; pregnant at 15 years; currently single, undergraduate]

I felt the world has ended, I felt I don't have anything again…I just felt nothing again would come to me that will be joy. I…I never felt encouraged at all. [Grace; pregnant at 16 years; currently single, completed junior secondary school]

Because of the anxiety about facing shame and stigma, some participants decided to leave home before the pregnancy became noticeable. A participant, one of the victims of sexual assault, was forced out of her uncle's home because of the hostility she faced as a consequence of her pregnancy.

…but I didn't want the baby…I didn't want them to see me with pregnancy, so my plan was to go far away from my house to make sure…I planned to go somewhere nobody knew me, [thinking] when I delivered the baby, I would just walk away. [Jummai; pregnant at 16 years; currently single, undergraduate]

I had to give birth in my uncle's house…It was shortly after I put to bed [gave birth], and then [a month later] I had to leave the house…Every day, they just kept reminding me [of] what happened, what I did…My uncle, too, at that period just built his house and they were about to pack [move] to their new house…It was Thursday, I was to write post-UME [university placement exam] on Friday, they told me on Thursday morning, so I was like, “can I just stay for this night so that I can go and write my exam, then after the exam I can pack [move]”? They said, “No, I just had to leave”.…That was how I left. [Monica; pregnant at 17 years; currently single, completed secondary education]

#### Moral dilemma: The decision to keep or terminate the pregnancy

Despite the difficulties associated with accepting and adjusting to pregnancy in adolescence, having an abortion was frequently not an initial option. Eight of the 17 participants reported having had a live birth and 3 who were pregnant at the time of this study had decided to keep the pregnancy. Two participants attempted to terminate their pregnancies but did not follow through for various reasons.

You know, I said I wanted to abort it, so I tried all the drugs I could…Then I changed my name because…it was at 3 months that I went for them to do the test. I even went to the hospital doctor, and they did everything, they said I should come back next [the following] week, and they balanced my diet. I now said ‘*kai’* [hey], I cannot…let me just stop because it is not the end of the world…so let me just keep it. [Binta; pregnant at 17 years; currently separated, completed junior secondary school]

…I said that let me do abortion…but I was afraid…hmm…so I did not do it [abortion]. [Laraba; currently pregnant at 16 years, single and cohabiting, completed junior secondary school]

Two participants had stillbirths. In addition, some participants reported terminating their pregnancies. Of these, one had a self-induced abortion, another reported terminating three pregnancies and the third participant reported terminating two pregnancies. One participant, upon the advice of her best friend, attempted an abortion, unsuccessfully, by drinking lime juice.

Well, the baby died in the…in the womb, the placenta and the baby separated. [Fatima; pregnant at 17 years; currently single, completed secondary education]

I did not find out [I was pregnant] until after 2 months but after I took something [self-induced abortion] and the reaction was…and I didn't understand what was happening…I wanted to go and use the toilet and I didn't even know that it was the baby that was coming out. It was very painful. [Patricia; pregnant at 17 years; currently single, undergraduate]

She [friend] gave me some hope because she told me that she heard people use lime to abort pregnancy, but she did not know at what month [gestation] it would work. Sometimes, she would go out and buy some lime for me. I felt a bit better then because I was hoping the lime would work. [Rifkatu; pregnant at 15 years; currently single, undergraduate]

For participants that initially considered an abortion but did not follow through, some decided against it themselves or their partner suggested that they should not terminate the pregnancy. The key reasons participants gave for not following through with getting an abortion included the fear of complications and death. Some believed it was morally wrong and sinful. Others were pressured by family and friends or partners to keep the pregnancy.

…I have already sinned; I should not go and add another sin. What if, in the process of doing that [abortion], I did not succeed, and end up dying, what will happen to me? [Kaneng; pregnant at 17 years; currently divorced, post-secondary education]

I…I…firstly, I prayed, and then I sought for other advice that will help me, I told my mom, my dad. They all said that I should not abort the baby, …that if I abort it now, that the consequences are…it will be much, …that the blood of the baby will be on their heads, and they will not be…be free, and…it will definitely bring them down. [Fatima; pregnant at 17 years; currently single, completed secondary education]

For one participant, the conditions surrounding the abortion plan made her reconsider her decision.

He [my boyfriend's cousin] took my boyfriend and me to a man who normally aborts children. So, it was at that man's place that I would stay until the baby was aborted…before I could go home or contact anyone…no contact with anyone until I lost the pregnancy. Nobody would know where I was. [Rifkatu; pregnant at 15 years; currently single; undergraduate]

#### Resilience: forging ahead despite the difficulties

Another subtheme that emerged from the adolescent pregnancy experience was resilience in the face of adversity to improve their social outcomes. Despite the initial shock and trepidation felt upon finding out about the pregnancy, some participants were able to refocus and move on with their lives.

I've tried to put the past behind me…It made me independent, I'm not dependent on anyone, nobody…down to my mom…nobody. I just know that I'll survive…I've learned to survive…even though I didn't finish school on time because of my pregnancy, I still feel I've come a long way. [Hannatu; pregnant at 15 and 18 years; currently single, completed university education]

…I picked the challenge, I moved from my family house [and now] live alone with the baby…I nurse her…I feed her. When she reached one year, I put her in a school…so I now said “I cannot end my life here only because I'm pregnant”…so I am now back to school…I have [found] something different doing, just a small business, that's what I'm taking care of myself with. [Kemi; pregnant at 18 years; currently single, undergraduate]

## Discussion

We explored the lived experiences of adolescent pregnancy in Jos, Nigeria. Participants lacked knowledge of SRH and parental communication on SRH. The lack of SRH knowledge doubtless contributed to contraceptive non-use and thus most pregnancies were unintended. The emotional toll of adolescent pregnancy on participants included feelings of fear and anxiety, some bordering on depression. Two main themes emerged from our findings: reflections on the meaning of adolescent pregnancy, as described by those who experienced it, and the adolescent pregnancy journey. The subthemes from the adolescent pregnancy journey included risk factors for adolescent pregnancy, the emotional toll of adolescent pregnancy, the dilemma around decision making to keep or terminate the pregnancy and resilience in forging ahead.

In reflecting on their experiences, participants described adolescent pregnancy as a negative and undesirable occurrence, one that they had not planned. These reflections are similar to those from recent studies in Nigeria where participants perceived teenage pregnancy negatively and associated it with serious consequences.^18,29^ The findings also suggest that while adolescents are having sexual relationships, they are mostly unprepared for the possibility of parenthood. These findings are congruent with those of a study in Ghana where adolescents who had been pregnant, in reflecting on a vignette about adolescent pregnancy, described the subject as ‘not ready or mature enough to handle the pregnancy and the baby’.^30^ Generally, teenage pregnancy has a negative connotation in several African cultures, including Nigeria,^29^ and this may explain why participants in our study attributed adolescent pregnancy to bad or immoral behaviour. Although adolescent pregnancy is perceived to be underage pregnancy, some participants thought it was permissible in the context of marriage. This perception may be due to the high prevalence of child marriage in low-resource countries and the expectation of childbearing soon after marriage.^8,9,14,15^

Many participants seemed to have a sense of what they wanted in their immediate future, and pregnancy was not part of that picture. Only one participant reported intentionality regarding a repeat pregnancy. These findings underscore the extent of the unmet need for contraception among adolescents. Several studies in different settings, including Nigeria and other resource-limited contexts, suggest that adolescents have significant unmet needs for contraception, increasing the burden of unintended pregnancy in this demographic group.^4,7,13^ Similar to the findings of other studies from Plateau State and South East Nigeria, some of the reasons for contraceptive non-use in this study included lack of SRH education at school and home, poor awareness of pregnancy risk and a lack of access to SRH advice or services before they became pregnant.^7,13^ Another reason for contraceptive non-use in this study was coercion and a lack of reproductive autonomy. This was evident among participants who relied on their partners to use a condom or practice the withdrawal method, even when they were aware of more effective contraceptive methods. More troubling were reports of coercion by some partners to have unprotected sex. Sexual assault and rape also contributed to adolescent pregnancy; of the 17 participants interviewed, 3 reported getting pregnant due to sexual assault. This is similar to findings from other studies in North East Nigeria and Ghana, where many adolescent pregnancies are attributed to sexual coercion and rape.^31,32^ Although the girl-child bears the brunt of the health and social consequences of adolescent pregnancy, she is not adequately empowered to make informed decisions on her SRH and others, notably her parents and partner, hold this decision-making power.^33^

There was a common misunderstanding that missed periods were a sign of STIs. We are not aware of any other study to report this finding in Nigeria. Further research can help us understand this misconception and identify effective ways of addressing it. Moreover, studies from Nigeria suggest that providing comprehensive sexuality education to adolescents can go a long way in helping them gain critical knowledge and skills to protect themselves from STIs and unintended pregnancy and to help them remain in school.^8,13^ Similarly, interventions that build the capacity of parents and caregivers of teenage children in parent–child communication on SRH would be beneficial.

Like ours, findings of past research in Nigeria highlight the emotional turmoil adolescents face when they become pregnant. Such turmoil is associated with cultural norms and their stage of emotional and physical development, combined with their inability to adapt quickly to the challenges and responsibilities that come with adolescent pregnancy discovery.^16,17^ Our findings also indicate that participants experienced feelings ranging from fear and guilt to self-condemnation for failing their families. Several accounts of fear and anxiety were related to having to discontinue their education or fear of how their parents, especially the father, would react to their pregnancy. Such feelings may be due to social norms and stigma surrounding adolescent SRH and adolescent pregnancy, similar to findings of studies involving adolescents in different parts of Nigeria.^14,34^ Congruent with findings of a study on the abortion-seeking behaviour of women in Nigeria,^35^ young persons are likely to be shamed for getting pregnant, especially outside marriage. In the current study, some participants decided to leave home, while some were sent away because of the ‘shame they had brought on themselves and the family’. Unresolved emotional stressors such as these could lead to more severe mental health problems in the future. Creating awareness of adolescent SRH and the social support needs of girls who become pregnant may contribute to reducing the stigma and related mental health outcomes during this critical period of development and transition to adulthood.

Deciding to keep or terminate an adolescent pregnancy is subject to many variables. Our findings indicate that it was a difficult decision for many young people. Although anxious about the difficulties and possible consequences they would face because of the pregnancy, similar to findings of previous research in Ghana,^31^ some participants in our study did not view abortion as a first option and many pregnancies resulted in a live birth. Studies in Nigeria and Ghana have shown that despite the initial shock and rejection of unintended adolescent pregnancies, most of the parties involved in decision making, notably the parents and the partner, pressure the adolescent into eventually accepting and keeping the pregnancy.^31,36^ The social stigma and criminalization of abortion in Nigeria may also contribute to the decision to keep the pregnancy. Although some participants considered getting an abortion, many did not follow through and ultimately kept the pregnancy, largely due to religious beliefs and fear of complications and possible death from the abortion. Abortion is illegal in Nigeria except to save the life of a woman and carries stiff penalties including jail time for both the woman and the provider.^36^ Nonetheless, in the current study there were accounts of pregnancy termination. These were unsafe abortions, indicating yet another difficulty with life-threatening implications imposed on pregnant adolescents.

Although participants who were still in school at the time they got pregnant dropped out, some were able to return to complete their secondary education and a few proceeded to higher education. This finding suggesting resilience of the young mother is not commonly included in the discourse on adolescent pregnancy and it warrants further research to understand the facilitators of returning to school after dropping out due to adolescent pregnancy. Outright expulsion or suspension are common reactions of educational institutions to adolescent pregnancy in Nigeria.^37^ While the Child's Right Act explicitly makes provision for girls who become pregnant to be allowed to return to continue their education, the policy still does not apply throughout Nigeria.^38^ Policy development and consistent implementation nationwide will be key drivers of successful interventions aiming to facilitate the return to school of pregnant adolescents. Several risk factors for adolescent pregnancy and subsequent educational interruptions can be ameliorated by providing comprehensive SRH education to adolescents in the education curriculum. Future studies with key stakeholders on the implementation and impact of the FLHE curriculum can inform education administrators on how to integrate the curriculum in a culturally sensitive and acceptable manner for successful scale-up in schools.

### Strengths and limitations

This study has limitations. We did not include the perspectives of parents and other stakeholders such as healthcare providers, educators and community members. The perspectives of other key stakeholders are valuable and can enhance our understanding of culturally acceptable ways of providing accurate SRH education to adolescents, both in school and at home, and would benefit future research. In addition, there is a possibility of selection bias by using the pregnancy crisis centre as the main recruitment site for this study. However, efforts were made to mitigate selection bias through snowball sampling and the use of personal and professional networks to recruit eligible youth who did not use the services of the crisis centre. Despite these limitations, the current study has strengths. By expanding our recruitment to include young adults in this study, we were able to learn how adolescent pregnancy influences the short-term outcomes of participants. For example, several years after an adolescent pregnancy, regrets over a lost childhood appeared to linger among participants. In addition, the findings of this study contribute to the evidence on resilience following adolescent pregnancy as well as the risk of repeat unintended pregnancies among those who had previously experienced adolescent pregnancy.

### Conclusions and policy recommendations

Adolescents who become pregnant face many important challenges. From participants’ reflections on the lived meaning of adolescent pregnancy through understanding their initial decision making and actions, findings from this study underscore the significance of adolescent pregnancy. Further, the current study indicates that adolescents lack access to accurate SRH education and services that can empower them to make informed decisions and take action for their physical and psychological health and well-being. There is not a single solution to the problem of adolescent pregnancy. Instead, we recommend multifaceted actions including empowerment through comprehensive sexuality education and awareness on SRH issues. Nationwide adoption, implementation and scale-up of the FLHE curriculum would be a good starting point. Similarly, national adoption of the provisions of the Child’s Right Act, with clear implementation guidelines, can provide a supportive framework to help adolescents return to school post-pregnancy. Education and awareness-raising interventions to reduce the stigma associated with SRH and pregnancy in adolescent girls would be beneficial in addressing social norms that prevent young persons from seeking crucial SRH services. In addition, interventions promoting youth-friendly services are warranted. Empowering parents and caregivers of adolescents to provide balanced sexuality education at home would complement the education received in school and help reduce the stigma associated with adolescent SRH issues.

## Data Availability

The data generated and analysed during the current study are not publicly available due to participant privacy and ethical restrictions of data sharing but are available from the corresponding author on reasonable request.
